# Molecular identification and antibiotic resistance pattern of actinomycetes isolates among immunocompromised patients in Iran, emerging of new infections

**DOI:** 10.1038/s41598-021-90269-5

**Published:** 2021-05-24

**Authors:** Hossein Ali Rahdar, Shahram Mahmoudi, Abbas Bahador, Fereshteh Ghiasvand, Fatemah Sadeghpour Heravi, Mohammad Mehdi Feizabadi

**Affiliations:** 1 Department of Microbiology, School of Medicine, Iranshahr University of Medical Sciences, Iranshahr, Iran; 2 Department of Parasitology and Mycology, School of Medicine, Iran University of Medical Sciences, Tehran, Iran; 3 Department of Medical Microbiology, School of Medicine, Tehran University of Medical Sciences, Tehran, Iran; 4 Department of Infectious Diseases, Imam Khomeini Hospital Complex, Tehran University of Medical Sciences, Tehran, Iran; 5 Surgical Infection Research Group, Faculty of Medicine and Health Sciences, Macquarie University, Sydney, Australia; 6 Thoracic Research Center, Imam Khomeini Hospital Complex, Tehran University of Medical Sciences, Tehran, Iran

**Keywords:** Antimicrobials, Bacteria, Clinical microbiology, Microbial communities, Pathogens, Microbiology

## Abstract

Recent advancements in DNA-based approaches have led to the identification of uncommon and rare bacterial pathogens. In this study, by utilizing a DNA-based approach, a total of 1043 clinical specimens were processed for the identification of actinobacteria targeting the 16S rRNA and *gyrB* genes. Drug susceptibility testing was also conducted using micro-broth dilution and PCR. Two isolates of *Nocardia flavorosea* and *Rhodococcus erythropolis* were reported for the first time in Iran. Also, *Nocardiopsis dassonvillei*, *Streptomyces olivaceus*, and *Streptomyces griseus* were reported for the first time in Asia. Infections caused by *Nocardia caishijiensis* and *Prauserella muralis* have also been reported in this study. The first Asian case of pulmonary infection caused by *Nocardia ignorata* and the first global case of brain abscess caused by *Nocardia ninae* and *Nocardia neocaledoniensis* have been reported in this study. Overall 30 isolates belonging to 6 genera (*Nocardia*, *Streptomyces*, *Rodoccoccus*, *Nocardiopsis*, *Rothia*, and *Prauserella*) were detected in 30 patients. All 30 isolates were susceptible to amikacin and linezolid. Three isolates including *Nocardia otitidiscaviarum* (n = 2) and *Nocardia flavorosea* (n = 1) were resistant to trimethoprim-sulfamethoxazole which were the first trimethoprim-sulfamethoxazole resistant clinical actinomycetes in Iran. Isolation of rare species of actinomycetes particularly *Nocardia* spp. requires urgent action before they spread clinically particularly among immunocompromised patients.

## Introduction

Actinomycetales order mainly known as aerobic actinomycetes are Gram-positive bacteria with a high guanine-plus-cytosine (GC) content in their genomes^1^. Bacterial classification based on cellular compositions such as meso or diaminopimelic acid, sugars, and long-range mycolic acid is the main method of classification in this group of bacteria^1^. Despite their low prevalence in clinical specimens, Actinomycetales can cause life-threatening infections in susceptible individuals like transplant recipients, patients receiving immunosuppressive drugs, and those with HIV, cancer, and diabetes, as well as in animals^2–7^. Depending on the site of entry and the host immune system, these pathogens may cause severe complications in affected individuals.

Actinomycetes infections may result in non-specific clinical symptoms such as granuloma and/or abscess formation^2–5^. Sample collection in actinomycetes-related infections is performed by invasive methods such as biopsy or bronchial wash^6,7^. Since these bacteria share several common phenotypic and chemical characteristics, molecular-based methods have been recently utilized for precise identification^8^.

Accurate identification and antibiotic susceptibility testing of actinomycete isolates especially from diffused infections and brain abscesses may help reduce mortality and the financial burden associated with actinomycetes-related infections as well as antimicrobial resistance^9,10^. Transformation of actinomycetes from an environmental saprophyte to a real pathogen is of primary importance particularly in vulnerable populations^11,12^. Due to the increased number of immunocompromised patients, opportunistic pathogens are the major leading cause of mortality in this group^4,5,13–17^. Fatal infections caused by these bacteria have been reported in immunocompetent individuals^9,18,19^. Treatment of these infections is a challenging approach and may last from months to years^4,11^. In this regard, treatment failure is also a common outcome and could result in the recurrence of the disease and death^20–22^.

The mortality associated with actinomycetal infections in transplant recipients and immunodeficient patients was reported as high as 3.5 times that of other bacterial infections^23,24^. Therefore, accurate and rapid identification of clinically important bacterial genera and species can help to manage the infection in a timely manner. Due to the paucity of information on the prevalence, species distribution, and drug-resistance of actinomycetes in Asia, particularly in developing countries such as Iran, we are aiming at providing useful information in this regard.

## Materials and method

### Study design and ethical statements

The present cross-sectional study was carried out at the teaching hospital of Tehran University of Medical Sciences, Tehran, Iran, from May 2017 to October 2019. This study was approved by the ethics committee of Tehran University of Medical Science (IR.TUMS.MEDICINE.REC.1397.261). All the experiments were performed in accordance with relevant guidelines and regulations. Informed consent was obtained for this study.

### Patients and specimens

A total of 1043 consecutive non-duplicate clinical samples including sputum, bronchoalveolar lavage (BAL), blood, drainage of the brain, or cutaneous abscesses and corneal scraping suspected to be actinomycetes infections were collected from patients in this study. The specimens were then transferred to the microbiology laboratory while observing standard safety protocols.

Recruited patients had the following inclusion criteria: (1) at least one underlying condition supporting infection by actinomycetes such as HIV infection, cancer, diabetes, autoimmune disorders, transplantation, immunodeficiency and chronic respiratory complications, (2) clinical or radiological findings consistent with a diagnosis of infection by actinomycetes such as lung consolidation, nodule, and cavitation in radiography, cutaneous lesions, solid and soft organ abscess. Patients with previous antibiotic therapy and those with evidence of tuberculosis or non-tuberculosis mycobacterial infections as well as fungal infections were excluded from the experiment.

### Isolation of actinomycetes

The direct microscopical examination was performed using gram and partial acid-fast staining. Specimens were cultured on blood and chocolate agar plates containing cycloheximide, vancomycin, and polymyxin B as well as antibiotic-free media^7,11,12^. After 3 weeks of incubation at 37 °C, suspicious colonies were selected for further investigation. Conventional biochemical tests including hydrolysis of hypoxanthine, tyrosine and xanthine, resistance to lysozyme, growth at 45 °C were performed and the colony morphology were assessed. Using these phenotypic tests, identification of *Nocardia* spp*.* at genus level was done^11,12^. Because phenotypic identification of all Actinomycetales is not feasible, suspicious colonies other than *Nocardia* were directly subjected to molecular identification.

### Molecular identification

Bacterial DNA was extracted using the boiling method^12^. In brief, PCR test was performed using primers 27F: 5′-AGA GTT TGA TCC TGG CTC AG-3′ and 1525R: 5′-AAG GAG GTG WTC CAR CC-3′^13^ targeting 16S rRNA gene as the primary target for identification. For rare *Nocardia* species, a fragment of *gyrB* gene, as a confirmatory target, was amplified and sequenced using primers F: 5'-CTT CGC CAA CAC CAT CAA CAC-3' and R: 5'-TGA TGA TCG ACT GGA CCT CG-3′^13^. PCR reactions were performed in a mixture of 25 μL containing 12.5 μL of the master mix, (Thermo Fisher Scientific, USA), 1 μL (0.2 µM) of each forward and reverse primers and 1 μL template DNA under following conditions: 5 min of preheating at 94 °C, 32 cycles of 30 s denaturation at 95 °C, 30 s of primer annealing at 58.5 °C, 1 min extension step at 72 °C and post cycling extension of 5 min at 72 °C. The process of amplification was carried out in a thermal cycler (Biorad Thermal Cycler, USA)^25^.

Electrophoresis of PCR products was performed in 1% agarose gel prepared in 1 × TAE buffer with 100 mV for 30 min. Initial quality assessment of PCR products (single and sharp bands) was performed using a UV transilluminator. The amplicons were subsequently sent for sequencing using both primers (Bioneer, South Korea). Results were trimmed and aligned with verified sequences in GenBank using the Basic Local Alignment Search Tool (BLAST). Standard databases for Nucleotide collection (nr/nt) and Highly similar sequences (megablast) program were used for alignment (https://blast.ncbi.nlm.nih.gov/Blast).

### Antimicrobial susceptibility testing

Drug susceptibility test was performed using the broth microdilution method in accordance with the CLSI M24-A2 guideline. Tested antibiotics were amikacin (1–64 μg/mL), amoxicillin-clavulanic acid (2/1–64/32 μg/mL), cefepime (1–32 μg/mL), ceftriaxone (4–64 μg/mL), ciprofloxacin (0.12–4 μg/mL), doxycycline (2–64 μg/mL), imipenem (2–64 μg/mL), linezolid (1–32 μg/mL), minocycline (1–8 μg/mL) and trimethoprim/sulfamethoxazole (TMP-SXT) (0.25/4.75–8/152 μg/mL). For isolates of *Rhodococcus* and *Rothia*, similar to previous studies^26–28^, different panels of antibiotics were tested. Serial dilutions of each antibiotic were made in 96-well microplates. Isolates were suspended in 200 μL of sterile water to prepare homogeneous suspensions of the bacteria. After adjustment to 0.5 McFarland standard turbidity, 50 μL of the solution was transferred into 10 mL of Mueller–Hinton broth and then added to each well of the micro-plate. After 48 to 72 h of incubation at 37 °C, MIC values were calculated and interpreted as susceptible (S), intermediate (I) or resistant (R) according to the CLSI^26^. *Nocardia transvalensis* NRRL B-10637 and *Nocardia asteroides* ATCC 19247 were served as control strains and incubated for 96 h for acceptable growth exhibition.

### Detection of TMP-SXT resistance genes (*sulf*, *int*, and *DfrA*)

**Figure 1:**
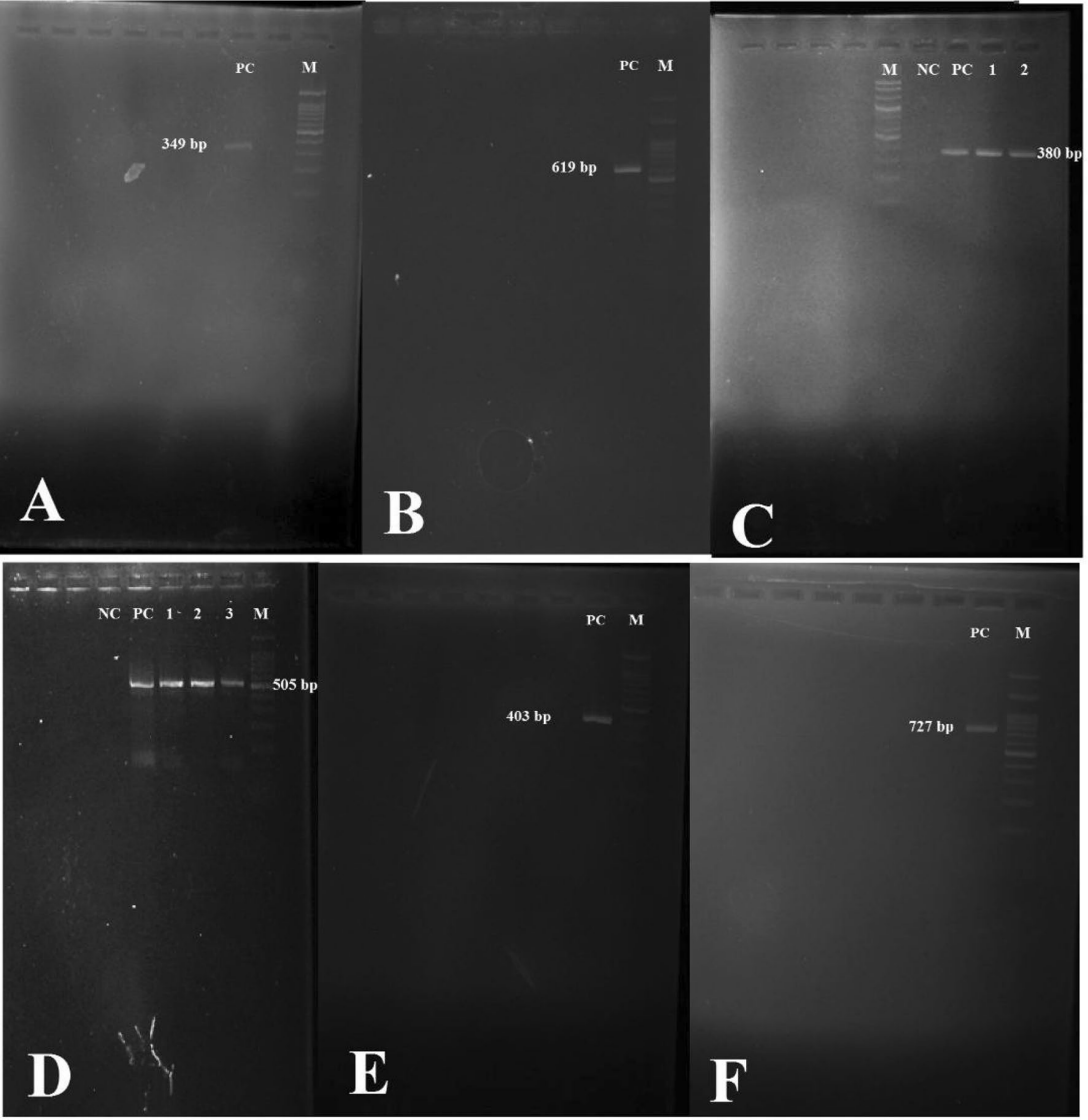
Gel electrophoresis of *dfrA* gene (panel **A**, PC: *Staphylococcus epidermidis* ATCC 12228), *sulf2* gene (panel **B**, PC: *Escherichia coli* strain AMR 130), *sulf1* gene (panel **C**, PC: *Escherichia coli* strain AMR 130, 1: *Nocardia otitidiscaviarum*, 2: *Nocardia flavorosea*), *int1* gene (panel **D**, PC: *Vibrio cholerae* O1 strain SK-10, 1: *Nocardia otitidiscaviarum*, 2: *Nocardia flavorosea*, 3: *Nocardia otitidiscaviarum*), *int2* gene (panel **E**, PC: *E. coli* strain having a R483), and i*nt3* gene (panel **F**, PC: *E. coli* strain having a pSMB731) PCR products. *M* Marker, *PC* positive control, *NC* negative control.

PCR was performed using specific primers to detect TMP-SXT resistance genes (*sulf1-sulf2, int1-int3*, and *DfrA*) as described previously^26^. The PCR products were visualized by electrophoresis in 1% agarose gel in 1 × TBE (Tris/Borate/EDTA) buffer, stained with safe stain load dye (CinnaGen Co. Tehran, Iran) under ultraviolet illumination. Results were interpreted based on the amplicon size as described previously^26^. To ensure the correctness of our results, *Staphylococcus epidermidis* ATCC 12228, *Vibrio cholerae* O1 strain SK-10, *E. coli* strain having a R483, *E. coli* strain having a pSMB731, and *E. coli* strain AMR 130 were used as positive controls for *dfrA*, *int1*, *int2*, *int3* and *sulf 1* and *2*, respectively (Fig. 1).

## Result

Based on the culture result, out of 1043 patients, 30 cases of actinomycetes infections (2.88%) were diagnosed. Of these, 22 (73.33%) and 8 (26.67%) were identified in males and females, respectively with a mean age of 55.43 ± 13.39 years old. Except for 2 healthy cases (6.67%), other cases had underlying medical conditions such as HIV/AIDS (n = 8), transplantation (n = 5), malignancy (n = 5), pemphigus vulgaris (n = 4), diabetes (n = 4) and corticosteroid therapy (n = 2). Involvement of the lungs was the most common form of infection (n = 24, 80%), followed by brain abscess (n = 3, 10%), cutaneous abscess (n = 1, 3.33%), arthritis (n = 1, 3.33%), and sepsis (n = 1, 3.33%). Two patients (6.67%) died of pulmonary infection and brain abscess before reporting the laboratory outcome.

According to patient history, trimethoprim/sulfamethoxazole (n = 19, 63.33%) was the most commonly prescribed antibiotic followed by imipenem (n = 6, 20%), trimethoprim/sulfamethoxazole and imipenem (n = 3, 10%), amikacin (n = 1, 3.33%), and levofloxacin (n = 1, 3.33%) in this study. Table 1 presents the baseline characteristics and clinical information of the patients.

**Table 1:** Baseline characteristics of patients, underlying conditions, laboratory findings, causative agents, treatment and outcome of 30 cases of actinomycetes infection.

No	Age/sex	Medical condition	Site of infection	Specimen	Direct smear	Growth at 45 °C	Hydrolysis of			Resistance to LYS	Molecular identification	GenBank accession number for *16s rRNA*	GenBank accession number for*gyrB*	Treatment	Outcome
							HYP	TYR	XAN						
1	39/M	HIV/AIDS	Lung	BAL	−	+	−	−	−	+	*Nocardia cyriacigeorgica*	KY817986	−	SXT	Cured
2	68/M	Liver transplantation	Lung	BAL	−	−	−	−	−	+	*Nocardia ignorata*	KY817987	MT739560	SXT	Cured
3	43/M	HIV/AIDS	Lung	BAL	−	-	NA	NA	NA	−	*Streptomyces olivaceus*	KY817988	MT739561	IMP	Cured
4	29/M	HIV/AIDS	Lung	BAL	−	-	NA	NA	NA	−	*Prauserella muralis*	KY817989	−	SXT	Cured
5	71/F	Healthy	Lung	BAL	−	+	−	−	−	+	*Nocardia cyriacigeorgica*	MH598412	−	IMP	Cured
6	39/M	Leukemia	Lung	Tracheal aspirate	−	-	+	+	+	-	*Nocardiopsis dassonvillei*	MH598413	MT739562	IMP	Cured
7	40/M	Pemphigus vulgaris	Lung	BAL	−	+	−	−	−	+	*Nocardia cyriacigeorgica*	MK680074	−	SXT	Cured
8	41/M	Pemphigus vulgaris	Lung	BAL	−	+	−	−	−	+	*Nocardia cyriacigeorgica*	MK680076	−	SXT	Cured
9	39/M	Pemphigus vulgaris	Lung	BAL	+	+	+	-	+	+	*Nocardia otitidiscaviarum*	MK680077	MT739563	SXT	Cured
10	58/M	Pemphigus vulgaris, diabetes	Lung	BAL	−	−	−	−	−	+	*Nocardia cerradoensis*	MK680078	MT739564	SXT	Cured
11	63/F	Corticosteroid therapy	Blood	Blood	−	−	−	−	−	−	*Rothia dentocariosa*	MK841031	MT739565	LEVO	Cured
12	55/M	Multiple myeloma	Lung	BAL	−	−	−	−	−	+	*Nocardia ignorata*	MK841035	MT739566	SXT	Cured
13	47/M	Corticosteroid therapy	Skin	Abscess drainage	−	−	+	+	−	+	*Nocardia brasiliensis*	MK841315	MT739567	SXT	Cured
14	34/F	Diabetes	Lung	BAL	+	−	−	−	−	+	*Nocardia asteroides*	MK841322	-	SXT	Cured
15	59/F	Liver transplant	Lung	BAL	−	+	−	+	−	+	*Nocardia neocaledoniensis*	MK841332	MT739568	SXT	Death
16	73/M	Healthy	Lung	BAL	−	+	+	−	+	+	*Nocardia otitidiscaviarum*	MK841414	MT739569	SXT	Cured
17	52/F	Multiple myeloma	Brain	Abscess drainage	−	+	+	−	+	+	*Nocardia otitidiscaviarum*	MK841475	MT739570	SXT + IMP	Death
18	69/M	Lung transplantation	Lung	BAL	−	−	−	−	−	+	*Nocardia caishijiensis*	MK875816	MT739571	SXT	Cured
19	74/F	HIV/AIDS	Lung	Sputum	−	−	−	−	−	+	*Nocardia asteroides*	MK875969	−	SXT	Cured
20	65/F	HIV/AIDS	Lung	BAL	+	+	−	−	−	+	*Nocardia kruczakiae*	MK876219	MT739572	SXT	Cured
21	63/M	Bone marrow transplantation	Brain	Abscess drainage	−	−	+	−	−	+	*Nocardia ninae*	MK876369	MT739573	SXT + IMP	Cured
22	74/M	HIV/AIDS	Lung	BAL	−	−	−	−	−	−	*Rhodococcus erythropolis*	MK876724	−	IM	Cured
23	61/F	Diabetes	Lung	BAL	−	+	−	−	−	+	*Nocardia cyriacigeorgica*	MK878397	−	SXT	Cured
24	51/M	HIV/AIDS	Brain	Abscess drainage	−	+	−	−	−	+	*Nocardia cyriacigeorgica*	MK878400	−	SXT + IMP	Cured
25	43/M	Lung transplantation	Lung	Tracheal aspirate	−	+	−	−	−	+	*Nocardia cyriacigeorgica*	MK878402	−	SXT	Cured
26	57/M	Diabetes	Lung	BAL	−	+	+	−	+	+	*Nocardia otitidiscaviarum*	MK878403	MT739574	AMK	Cured
27	65/M	Multiple myeloma	Lung	BAL	−	−	−	−	−	+	*Nocardia asteroides*	MK878404	−	SXT	Cured
28	47/M	HIV/AIDS	Lung	Tracheal aspirate	−	+	−	−	−	+	*Nocardia flavorosea*	MK878408	MT739575	SXT	Cured
29	73/M	Diabetes	Joint	Synovial fluid	+	−	NA	NA	NA	−	*Streptomyces griseus*	MK878410	−	IMP	Cured
30	71/M	Leukemia	Lung	BAL	−	−	+	+	+	−	*Nocardia dassonvillei*	MK878413	MT739576	IMP	Cured

*M* male, *F* female, *BAL* bronchoalveolar lavage, *HYP* hypoxanthine, *TYR* tyrosine, *XAN* xanthine, *LYS* lysozyme, *SXT* trimethoprim/sulfamethoxazole, *IMP* imipenem, *LEVO* levofloxacin, *AMK* amikacin, *NA* not applicable.

**Figure 2:**
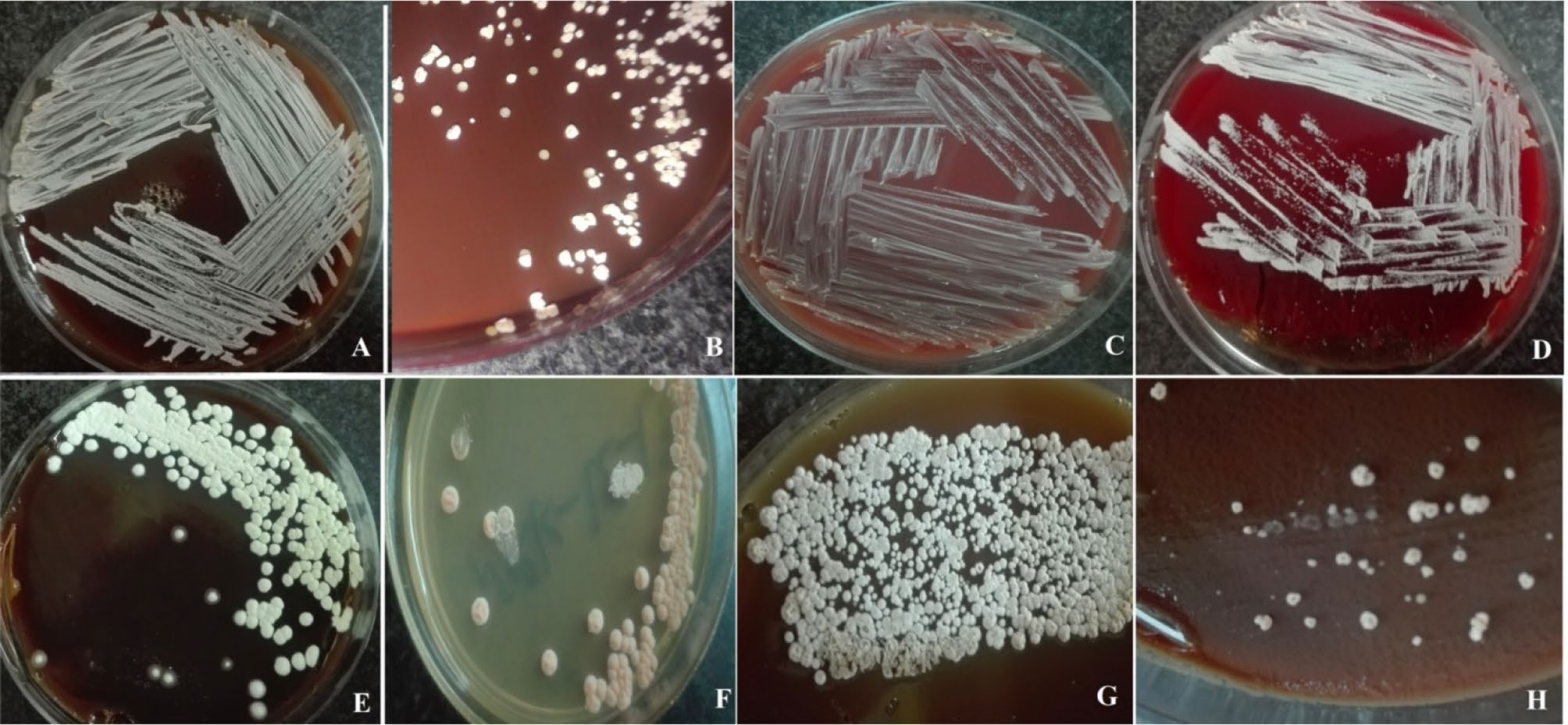
Pure colonies of isolated species (**A**) *Nocardia cyriacigeorgica,* (**B**) *Nocardia kruczakiae*, (**C**) *Nocardia flavorosea,* (**D**) *Nocardia asteroides*, (**E**) *Streptomyces griseus*, (**F**) *Nocardia cerradoensis*, (**G**) *Nocardia caishijiensis*, (**H**) *Prauserella muralis.*

**Figure 3:**
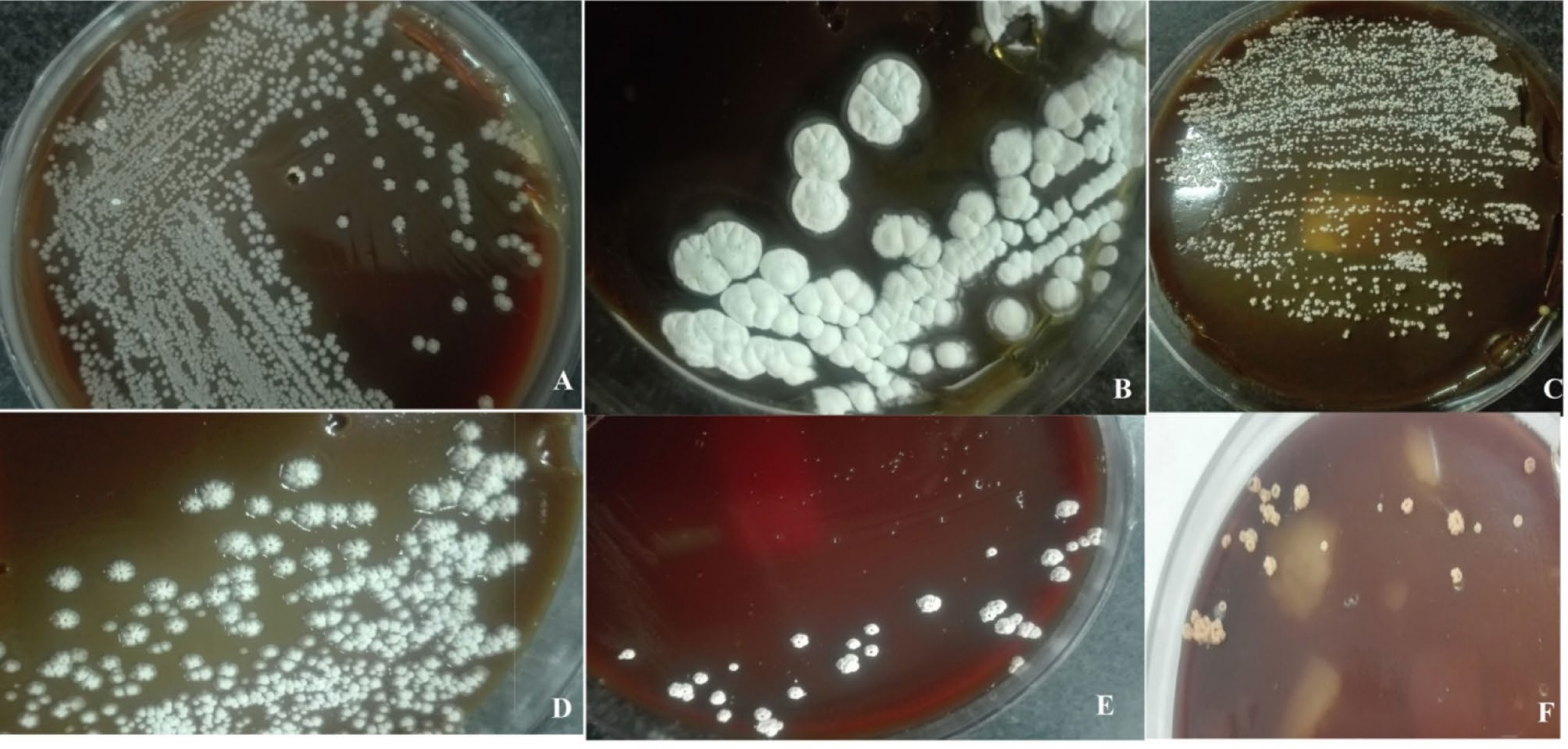
Pure colonies of isolated species (**A**) *Nocrdia otitidiscaviarum*, (**B**) *Nocardiopsis dassonvillei* (**C**) *Nocardia cyriacigeorgica*, (**D**) *Nocardia ninae,* (**E**) *Nocardia.brasiliensis*, (**F**) *Rhodococcus erythropolis.*

According to the DNA-based method in this study, the causative agent of 30 cases of actinomycetes belonged to 6 genera (*Nocardia*, *Nocardiopsis*, *Streptomyces, Prauserella*, *Rhodococcus*, and *Rothia*) and 17 species with the dominance of *Nocardia cyriacigeorgica* (n = 7, 23.33%), followed by *Nocardia otitidiscaviarum* (n = 4, 13.33%), *Nocardia asteroides* (n = 3, 10%), *Nocardia ignorata* (n = 2, 6.67%), *Nocardiopsis dassonvillei* (n = 2, 6.67%) and 1 isolate (3.33%) from 12 other species including rare pathogens such as *Prauserella muralis*, *Rhodococcus erythropolis*, and *Rothia dentocariosa* (Table 1; Figs. 2, 3).

All *Nocardia* isolates (n = 23) were susceptible to amikacin and linezolid, while some were non-susceptible (resistant/intermediate) to trimethoprim-sulfamethoxazole (n = 3, 13.04%), imipenem (n = 6, 26.08%), minocycline (n = 6, 39.13%), ceftriaxone (n = 11, 47.83%), doxycycline (n = 11, 47.83%), cefepime (n = 14, 60.87%), amoxicillin-clavulanic acid (n = 18, 78.26%), and ciprofloxacin (n = 20, 86.96%). *Nocardiopsis dassonvillei* isolates (n = 2) were non-susceptible to amoxicillin-clavulanic acid (50%), doxycycline (50%), ciprofloxacin (100%), ceftriaxone (100%) and cefepime (100%) but susceptible to other antimicrobial drugs.

*Streptomyces olivaceus* and *Streptomyces griseus* were non-susceptible to 3 and 4 out of 10 tested antimicrobial drugs, respectively. Regarding the rare species *Prauserella muralis*, *Rhodococcus erythropolis*, and *Rothia dentocariosa*, a non-susceptible phenotype was observed to 2 out of 10, 5 out of 11 and one out of 8 tested antimicrobial drugs, respectively. Susceptibility pattern of all isolates is presented in Table 2.

**Table 2:** Antimicrobial susceptibility pattern of actinomycetes isolates in this study.

Species	N	Resistance/Intermediate (%)																			
		IPM	SXT	MIN	AMC	AMK	LZD	CIP	CRO	FEP	DOX	CFT	P	GM	E	AMP	PIP	CFZ	CAZ	CAX	LEV
*Nocardia cyriacigeorgica*	7	0	0	14.3	71.5	0	0	100	14.3	28.6	28.6	−	−	−	−	−	−	−	−	−	−
*Nocardia otitidiscaviarum*	4	100	50	50	100	0	0	100	100	100	50	−	−	−	−	−	−	−	−	−	−
*Nocardia asteroides*	3	0	0	100	100	0	0	100	100	100	33.3	−	−	−	−	−	−	−	−	−	−
*Nocardia ignorata*	2	0	0	0	100	0	0	100	100	100	50	−	−	−	−	−	−	−	−	−	−
*Nocardia cerradoensis*	1	0	0	100	0	0	0	100	0	0	100	−	−	−	−	−	−	−	−	−	−
*Nocardia neocaledoniensis*	1	0	0	100	100	0	0	100	0	100	100	−	−	−	−	−	−	−	−	−	−
*Nocardia brasiliensis*	1	100	0	0	0	0	0	100	100	100	100	−	−	−	−	−	−	−	−	−	−
*Nocardia kruczakiae*	1	0	0	0	0	0	0	100	0	0	0	−	−	−	−	−	−	−	−	−	−
*Nocardia flavorosea*	1	100	100	100	100	0	0	0	0	0	100	−	−	−	−	−	−	−	−	−	−
*Nocardia caishijiensis*	1	0	0	0	100	0	0	0	0	0	0	−	−	−	−	−	−	−	−	−	−
*Nocardia ninae*	1	0	0	0	100	0	0	0	0	100	100	−	−	−	−	−	−	−	−	−	−
*Nocardiopsis dassonvillei*	2	0	0	0	50	0	0	100	100	100	50	−	−	−	−	−	−	−	−	−	−
*Streptomyces olivaceus*	1	0	0	0	0	0	0	0	100	100	100	−	−	−	−	−	−	−	−	−	−
*Streptomyces griseus*	1	0	0	0	100	0	0	100	100	0	100	−	−	−	−	−	−	−	−	−	−
*Prauserella muralis*	1	0	0	0	0	0	0	100	0	100	0	−	−	−	−	−	−	−	−	−	−
*Rhodococcus erythropolis*	1	0	−	100	−	100	0	−	100	100	100	0	0	0	0	−	−	−	−	−	−
*Rothia dentocariosa*	1	0	−	−	−	−	−	−	−	0	−	−	−	−	−	0	0	100	0	0	0

*IPM* imipenem, *SXT* trimethoprim-sulfamethoxazole, *MIN* minocycline, *AMC* amoxicillin-clavulanic acid, *AMK* amikacin, *LZD* linezolid, *CIP* ciprofloxacin, *CRO* ceftriaxone, *FEP* cefepime, *DOX* doxycycline, *cft* cefotaxime, *P* penicillin, *GM* gentamicin, *E* erythromycin, *AMP* Ampicillin, *PIP* piperacillin, *CFZ* cefazolin, *CAZ* ceftazidime, *CAX* ceftriaxone, *LEVO* levofloxacin.

Due to the common prescription of trimethoprim/sulfamethoxazole in actinomycetes infections, especially nocardiosis, resistant isolates to this antibiotic were further studied. A total of 3 isolates including 2 *Nocardia otitidiscaviarum* and one *Nocardia flavorosea* isolates were resistant to trimethoprim/sulfamethoxazole. Genes *int1*, *sulf1* were detected in all three isolates (Fig. 1).

## Discussion

Actinomycetes can be isolated from different environmental sources including soil, water, decaying plants, and animals^11,12^. Unlike many bacterial communities including *Bacillus* and *Clostridium* which produce endospore as a dormant structure, in actinomycetes, mycolic acid and a peptidoglycan layer form a non-productive and tough structure to survive under harsh conditions for a long period of time^29–32^.

Immunodeficiency, transplantation procedure, and old age predispose individuals to bacterial infection particularly caused by opportunistic pathogens. Meanwhile, with the exception of a few studies, a comprehensive study on the evaluation of actinomycetes is not available. On the other hand, traditional and culture-based methods have inadequacies in the detection of unexpected and rare pathogens such as actinomycetes in clinical specimens. Therefore, further investigation using sequencing-based approaches is required to identify actinomycetes at species level accurately and evaluate antibiotic resistance patterns in order to prevent the progression of infection and extend the current knowledge regarding the epidemiology of infection^13–17,33^.

The prevalence of actinomycetes infections varies geographically. In this study, the prevalence of actinomycetes infections was 2.88% (30 out of 1043). The prevalence of *Nocardia* infections was 2.21% (23 out of 1043) which was slightly higher than the estimated prevalence of nocardiosis in Iran (1.88%)^34^. From 1970 to 2005, the prevalence of nocardiosis in Asia has been estimated to range between 0.001% in the United Arab Emirates to 1.9% in China, while in Africa, it was estimated to be 1.8% in Congo to 4.1% in Nigeria^34^. However, due to the paucity of information in this regard, almost all the recent studies were focused on previously isolated actinomycetes or retrospective reviews of medical records and were unable to provide a comprehensive prevalence of the infection. Similar prospective studies are needed to improve our understanding of the exact prevalence of actinomycetes in different countries.

In this study, pulmonary infection (n = 24, 80%) was the most common complication among patients and was higher than previous reports in Iran (50%)^13^, the United States (57.4%) and China (65.22%). Extrapulmonary infection was found in 6 patients, of them 3 were diagnosed with brain abscess. The brain abscesses made 10% of all cases in this study and is close to the previous reports in Iran (9%)^13^. Based on these results, nocardiosis should be considered in all patients with pulmonary infections as the lungs are the most common site of infection. However, extrapulmonary infections, especially brain abscesses should not be overlooked. Although the mortality associated with brain abscesses is high (40–50%), it can also be misdiagnosed with neoplasms, fungal or mycobacterial abscesses^19^.

Due to the immune deficiency and low CD4+ count, HIV patients are prone to actinomycetes infections. In this study, HIV/AIDS was the most common underlying condition which was found in 8 patients (26.67%) and was consistent with the previous study in Iran^13^. Delayed diagnosis and treatment of actinomycetes infections among HIV patients can lead to a high mortality rate from 18 to 66%^35^.

Less severe diseases can also predispose patients to actinomycetes infections. Diabetes was the most common underlying condition reported in a study conducted in China^36^. Other underlying conditions such as transplantation, malignancy, and corticosteroid therapy can also predispose patients to actinomycetes infection. Healthy individuals are also at risk of actinomycetes infection, as 6.67% of patients in this study did not have any underlying disease. Similarly, Hashemi-Shahraki et al. and Yi et al. have diagnosed 21.1% and 44.1% of actinomycetes infections in their studies among healthy individuals, respectively^13,36^. Therefore, in the presence of relevant symptoms, actinomycetes infections should be considered in the differential diagnosis, not only in immunocompromised patients but also in immunocompetent individuals.

*Nocardia cyriacigeorgica* (n = 7, 23.33%) followed by *Nocardia otitidiscaviarum* (n = 4, 13.33%) were the most common identified species in this study which was not in agreement with the previous study in which *Nocardia asteroides* (n = 31, 24.41%) and *Nocardia cyriacigeorgica* (n = 25, 19.69%) were the most common isolated species^13^.

Our findings are not in agreement with other countries. For instance, a study conducted on hematopoietic stem cell transplant recipients in Japan^37^ revealed the *Nocardia farcinica* (n = 3, 42.86%) as the leading species, similar to studies conducted in China with 42.1%^40^ and 34.78%^38^ of cases caused by this pathogen. However, in the United States^39^ and Spain^40^, *Nocardia nova* has been reported as the most common species contributing to 28% and 29.57% of cases, respectively. Therefore, molecular-based studies providing more precise results are needed in all countries for the identification of common and uncommon species in actinomycetes infections. Using molecular-based approaches, more unexpected and rare bacterial species are expected to be detected in similar cases.

In the present study, infections due to a set of rare actinomycetes were identified. To the best of our knowledge, in this study, *Nocardia flavorosea* and *Rhodococcus erythropolis* were reported for the first time in Iran, and *Nocardiopsis dassonvillei*, *Streptomyces olivaceus*, and *Streptomyces griseus* for the first time in Asia, and infections caused by *Nocardia caishijiensis* and *Prauserella muralis* were reported for the first time in the world. We also reported the first Asian case of pulmonary infection caused by *Nocardia ignorata* and the first global cases of brain abscess cause by *Nocardia ninae* and *Nocardia neocaledoniensis* in this study.

From the treatment point of view, trimethoprim/sulfamethoxazole has been the most common antibiotic prescribed in actinomycetes infections, particularly nocardiosis. In the present study, 3 isolates (10.71%) (2 *Nocardia otitidiscaviarum* and 1 *Nocardia flavorosea*) were resistant to trimethoprim/sulfamethoxazole. By excluding non-*Nocardia* isolates, this rate was 13.04% among *Nocardia* species which was higher than the previous report in Iran (1.57%)^13^. The rate of trimethoprim/sulfamethoxazole resistance varies in different studies. While all isolates were susceptible to this antibiotic in one study^36^, other studies have reported resistance rates of 2%^41^, 16.12%^42^, 21.74%^38^, and 42%^39^.

Because of a high rate of the susceptibility of *Nocardia* species to linezolid and amikacin, both antibiotics have been prescribed to treat the infections caused by these organisms^13^. High susceptibility of isolates to linezolid in this study may imply the effectivity of this antibiotic in similar cases. Also, all *Nocardia* isolates were susceptible to amikacin and only *Rhodococcus erythropolis* isolates (3.45%) were resistant to this antibiotic. Three trimethoprim/sulfamethoxazole-resistant isolates were susceptible to linezolid and amikacin in this study demonstrating the application of linezolid and amikacin in infections caused by trimethoprim/sulfamethoxazole-resistant isolates. However, it is worth pointing out that there are also few studies reporting amikacin and linezolid resistance^40,42,43^.

Also, the *int1* and *sulf1* were detected in all trimethoprim/sulfamethoxazole-resistant isolates which were consistent with previous findings^26^. Regarding the non-*Nocardia* species, the susceptibility patterns were species-specific. In general, there was no infection with imipenem, trimethoprim/sulfamethoxazole, and linezolid resistance isolates. However, resistance to amikacin was observed in the *Rhodococcus erythropolis* isolate and with the exception of cefazolin, the *Rothia dentocariosa* isolate was susceptible to the all tested antibiotics.

Based on our findings, there is an urgent need for proper identification and antibiotic susceptibility testing of actinomycetes isolates in all regions particularly in vulnerable patients to actinomycetes infections.

## Conclusion

Based on the results, a wide range of actinomycetes species can lead to severe infections in vulnerable patients. Although *Nocardia* was reported as the leading genus in this study, emerging new infections due to *Nocardia ignorata*, *Nocardiopsis dassonvillei, Nocardia ninae*, *Rhodococcus erythropolis*, *Nocardia kruczakiae*, *Nocardia flavorosea, Streptomyces griseus*, *Nocardia cerradoensis*, *Nocardia caishijiensis*, *Prauserella muralis* were also identified. Inter-species differences were observed in the antimicrobial resistance pattern in isolates harboring trimethoprim/sulfamethoxazole-resistance genes. Precise identification of actinomycetes isolates using molecular-based approaches is required for a better understanding of the epidemiology of infection. Although accurate identification of this group of bacteria is challenging in many laboratories, evaluation of this pathogen particularly in patients with underlying medical conditions should not be overlooked. Furthermore, the evaluation of the antibiotic resistance pattern of actinomycetes isolates should not be limited to a period of time and continuous monitoring is required for the prevention of infection and the improvement of therapeutic approaches.

## Data Availability

The sequencing data are accessible in the Sequence Read Archive (SRA)/NCBI (http://www.ncbi.nlm.nih.gov/sra) under accession numbers specified in Table 1.
